# SPARK Resilience in the workplace: Effectiveness of a brief online resilience intervention during the COVID-19 lockdown

**DOI:** 10.1371/journal.pone.0271753

**Published:** 2023-03-15

**Authors:** Ilona Boniwell, Evgeny Osin, Larissa Kalisch, Justine Chabanne, Line Abou Zaki

**Affiliations:** 1 Positran, Épone, France; 2 School of Psychology, University of East London, London, United Kingdom; 3 International Laboratory of Positive Psychology of Personality and Motivation, HSE University, Moscow, Russia; 4 Laboratory LINP2, University of Paris Nanterre, Nanterre, France; 5 School of Psychology and Sports Science, Anglia Ruskin University, Cambridge, United Kingdom; Public Library of Science, UNITED STATES

## Abstract

Science asserts that resilience at work can be developed, with evidence pinpointing to multiple resources that can be built through deliberate coaching, training and interventions. This paper presents a mixed-methods study exploring the effectiveness of group coaching using SPARK Resilience training, a model and a structured coaching protocol that have been administered in educational and workplace settings in face-to-face format and remotely. The study used a non-randomised controlled design with a pre-test and a post-test in a sample of French adults (*N* = 101 in the intervention group and *N* = 86 in the waitlist control group). The SPARK Resilience programme was administered online with 8 sessions spanning 4 weeks in April 2020, during the very early stage of the pandemic and lockdown in France. The results indicate beneficial effects of the intervention on meaning, resilience, positive affect, and perceived stress outcomes (*d* in the .40-.56 range), as well as weaker effects on negative affect (*d* = .35) and work engagement (*d* = .21). Moderator analyses suggest that the effects of the intervention on perceived stress and negative affect tended to be stronger for older adults. Participants reported high levels of satisfaction with the intervention and provided 151 responses to three open-ended questions that were coded using thematic analysis, revealing specific benefits of the intervention. The findings are interpreted within the pandemic context, showing the way resilience interventions can help people overcome unprecedented challenges.

## Introduction

### Mental health and the COVID pandemic

Mental health issues were already of concern for organisations before the COVID-19 pandemic. Adult Psychiatric Morbidity Survey 2014 conducted in a large and representative British sample has positioned the overall prevalence of mental health problems in adults over 16 years of age at 18.9%, an increase from 17.6% in 2007 [1]. An earlier study in a representative French sample has found a 32.4% prevalence of mental disorders in the adult population [2]. An influential review [3] has concluded that around 15% of people at work suffer from an existing mental health condition, stating that the prevalence of common mental health problems has increased over the last two decades, with the biggest rises in anxiety and depression, particularly among younger women and older men.

Mental health pressures have intensified since the beginning of 2020. Declared a global pandemic by the World Health Organization (WHO), COVID-19 led many countries to pronounce a state of emergency, issue restricted movement orders and, in most cases, impose lockdowns. Workwise, although some work patterns have intensified (especially in the healthcare sector: [4]), many jobs have been put on standby or shifted towards remote work. COVID-19 has brought with it pandemic-specific stressors, such as new perceived threats to health and safety, risk of contagion, information overload, quarantine and confinement, stigma, social exclusion, long periods of isolation, as well as financial losses and job insecurity. According to research, different workplace factors, such as occupational role, health and safety management, teleworking, as well as government support, could either mitigate or aggravate the mental health issues during the pandemic [5, 6].

Data from the UK Household Longitudinal Study based on nearly 9,000 British adults indicate that psychological distress increased among all employees from 20.1% at the 2017–2018 baseline to 31.8% in April 2020 with prevalence increasing similarly across all demographic groups over time. Furthermore, distress increased from 23.7% at baseline to 69.4% in April 2020 for those who were permanently laid off [7]. Longitudinal studies conducted in France confirm the negative effects of lockdown on mental health, revealing a stronger impact on women, younger adults (aged 23–49), and the elderly (aged over 70) [8].

### The concept of resilience

Many employees are able to deal with stressors individually. However, an accumulation of multiple stressors as well as an extended duration of exposure to stress could lead to a detrimental impact on the psychological and physical well-being of employees, as well as on their performance at work [9, 10]. The construct of workplace resilience has been proposed to describe a system of factors that could serve as a buffer against stress and the negative side effects of job demands. It was described in terms of cognitive and behavioural capabilities associated with responding to adversity in adaptive ways, seeking and utilizing opportunities in work challenges [11].

The concept of resilience was conceived about 40 years ago when researchers noticed that some people manage to adapt well to life despite the presence of high-risk circumstances (such as losing parents young, for example). This step was a positive divergence from the typical pathological models based on the assumption that early traumatic experiences undoubtedly result in negative life outcomes. However, the scientific research devoted to this phenomenon was scarce at the time and it is only in the past 20 years that the investigation of resilience has expanded considerably. A recent review revealed that the usage of the term ‘resilience’ in the academic literature has increased eightfold in the last two decades [12].

Resilience is generally defined in functional terms, as the capacity for successful adaptation and/or growth in the face of significant adversity [13–15]. It is a multi-faceted construct, being both conceptualized as a capacity to bounce back from negative emotional experiences and as an active dynamic process reflecting a person’s flexibility in response to changing situational demands. Within this general definition, two facets can be distinguished: recovery, referring to a capacity to regain one’s psychological, physiological, and social equilibrium after stress, and resistance, which refers to the capacity to withstand the challenge of stress and to carry on pursuing one’s goals in the face of adversity [16, 17].

Research indicates that higher levels of self-reported resilience are associated with lower levels of psychological distress, anxiety, and depression symptoms in various age groups and that these associations are stronger in individuals facing adversity [18]. Workplace resilience outcomes include higher job performance, mental and physical health, better relationships, and more positive work-related and change-related attitudes [9, 19, 20].

### Team resilience

Resilience is not a single trait, but, rather, a function supported by a whole range of resources that exist both at the individual level (psychological and physiological resources) and the level of the environment (community, team, organisation resources, etc.) [11, 14, 16]. The antecedents of workplace resilience include personality traits and cultural value orientations [20], the tendency to appraise situations as challenges rather than as threats [21]; self-regulation [10]; positive affect [21]; self-efficacy [22]; personal values and meaning [23]; openness to learning within the organisational culture [24], relationship with line manager [25], social competences and social support more generally [25], as well as some transformational and transactional leadership dimensions [26].

In recent years, research has started to focus on resilience as a collective phenomenon, given that work is increasingly structured in and around teams. A team is defined as a group of interdependent persons who share the responsibility for a common outcome [27]. Modern-day working environments expose their teams to a variety of stressors, such as tight deadlines, frequently changing team structures, the necessity to accomplish a larger number of tasks with fewer resources, and, finally, important consequences of the shared project’s outcome in terms of its financial or psychological impact for the team members [28].

Team resilience can be defined as a “*dynamic*, *psychosocial process which protects a group of individuals from the potential negative effect of stressors they collectively encounter*. *It comprises of the processes whereby team members use their individual and collective resources positively to adapt when experiencing adversity”* [24 p45]. Studies have found that resilient teams are more creative, productive, and flexible during tough times [29]. Simultaneously, team members display a higher level of well-being and more readiness for future challenges [30].

Although team resilience is undoubtedly influenced by individual factors such as personal knowledge, skills, diversity and values, it tends to be more dependent on team social and process factors [31], as well as organisational factors [32]. Stoverink and colleagues [33] identify four antecedents of team resilience, notably, the mental model of teamwork, the capacity to improvise, psychological safety, and team potency. The latter factor is akin to collective efficacy which has been found to play a key role in team resilience, as it reflects the team members’ belief in their capacity to face challenges together [31]. Another important factor is team members’ resourcefulness which enables them to get to know each other and build on their strengths in tough times [34]. Social identity, or a fusion of individual identities into a collective one by thinking, feeling, and behaving in common ways that foster ingroup membership, has also been identified as a contributor to team resilience [31]. McEwen & Boyd [30] have identified a number of similar factors, including also perseverance and capability (i.e. continuously seeking feedback), otherwise termed team learning orientation [29].

### SPARK Resilience programme

There is evidence that the psychological mechanisms of workplace resilience can be developed by means of structured training and workshops using a wide variety of techniques identified in the literature [20, 35–38]. One notable example of a large-scale intervention is the Master Resilience Training implemented in the American army enhancing the perceived resilience, mental health, and adaptive behaviours of the personnel [39, 40].

A recent meta-analysis of data from 1,584 samples indicates small to moderate efficacy (Hedges’ *g* = 0.48) of resilience interventions, in particular, against performance, emotional, and symptom outcomes [41]. However, some reviews suggest that the studies of workplace resilience interventions vary in quality, design, and implementation [19] and that the effects of these interventions on health and performance outcomes measured in a longer term (over 1 month) are substantially weaker (*d* = 0.07) [9].

One of the multiple resilience intervention protocols established and tested in the last two decades is the SPARK Resilience Programme (RP), which was originally developed as a universal school-based resilience curriculum based on cognitive-behavioural therapy and positive psychology interventions [42]. Pluess and Boniwell [43] conducted a study in the UK with 11-year-old secondary school students (*N* = 363) to find out whether the personality trait of Sensory-Processing Sensitivity would moderate the efficacy of the SPARK RP aimed at the prevention of depression. Adolescents with moderate to high sensitivity showed a significant increase in self-esteem scores and a decrease in depression scores; both effects were sustained at a three-month follow-up. A further study explored the effects of the SPARK program on depression symptoms and resilience in a high-risk population of 11 to 13-year-old students in England (*N* = 438) [44]. The study found a decrease in depression symptoms immediately after the intervention and at a six-month follow-up. Resilience scores were also significantly higher in the treatment cohort compared to the control cohort at post-treatment and follow-up assessments. Since then, SPARK Resilience has been extensively implemented in the UK, France, Netherlands, Japan, and Singapore. A recent study from Japan with 407 high school students has found that the program was effective at enhancing students’ overall self-efficacy and that highly sensitive students, who had had significantly lower well-being scores at baseline than their counterparts, responded more positively to the intervention, showing a greater reduction in depression and promotion of self-esteem [45].

These findings showing the efficacy of the SPARK Resilience Programme in schools have led to the development of a new variant of the program aimed at employees and called *SPARK Resilience in the Workplace*. This, more recent, intervention protocol uses the same organizing framework informed by cognitive-behavioural therapy, but integrates the latest research evidence on workplace resilience at individual and team levels and uses a wider range of interventions adapted for adult populations. In response to the organisational demands associated with the COVID-19-related lockdown, this version of the protocol has been adapted for synchronous online delivery and tested for the first time in the present study using a community sample of volunteer participants coming from employed, self-employed, and student populations.

### SPARK Resilience in the workplace intervention protocol

**Fig 1:**
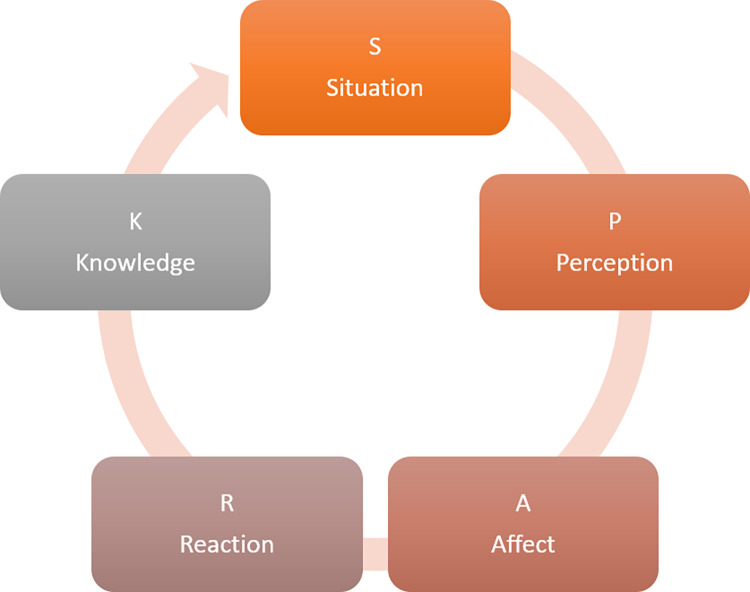
The SPARK Resilience model.

The development of the SPARK model was informed by the original ABC model of Albert Ellis [46]. Organised around the SPARK acronym, the model breaks down the responses to stressful situations into five components: Situation, Perception, Autopilot, Reaction, and Knowledge (Fig 1). Everyday **S**ituations, as a function of individual **P**erceptions, tend to trigger an emotion or **A**ffect (i.e., automatic emotional responses). This leads to subsequent behavioural **R**eactions and learning, or **K**nowledge gained from the experience. To enhance resilience in the same **S**ituation, it is important, first, to view it as a collection of neutral facts, to challenge one’s **P**erception of adversity, to notice and regulate one’s automatic **A**ffective responses, and to control one’s negative behavioural **R**eactions. This usually leads to an enhanced **K**nowledge, or understanding of the situation and one’s role in it.

Whilst the SPARK model can be used as a coaching tool in itself, its most important role is that of an organising framework that could help structure and introduce multiple strategies and tools aimed to enhance resilience. Each of the SPARK factors is used to introduce relevant resilience-enhancing strategies, enabling participants to experiment with over twenty-five tools and practices issued from scientific sources [10, 21–25, 35–38].

**Fig 2:**
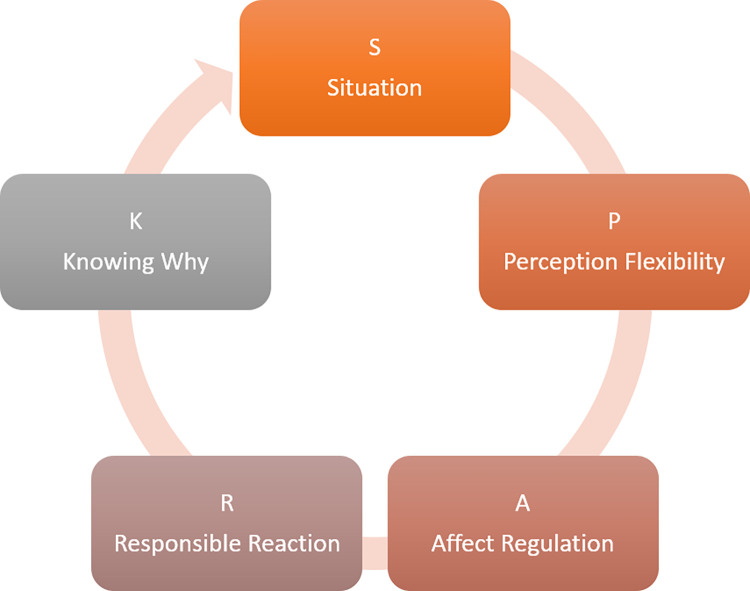
The SPARK solutions model.

The intervention protocol (see the session themes listed in Table 1) starts with an introduction and peer coaching around the SPARK Resilience model and then progresses onto resilience skills, organised around the SPARK Solutions model (Fig 2). Whilst no specific skills associated with S are introduced, Sessions 2 to 5 are structured around exploring and practicing cognitive skills (termed Perception Flexibility for ease of remembering), emotional regulation skills (Affect Regulation), behavioural skills (Responsible Reaction), and meta-cognitive skills (Knowing Why) using a variety of evidence-based strategies.

**Table 1:** SPARK Resilience in the workplace sessions and associated tools.

Session	Strategies
1. Let’s SPARK	SPARK Resilience model
2. Perception flexibility	Disputation Distancing Re-framing De-catastrophising Cognitive defusion
3. Affect regulation	Affect labelling Disclosure Flow Mindfulness Sleep, exercise and nutrition
4. Responsible reaction	Active avoidance Exposure Social connections Assertiveness Goal orientation
5. Knowing why	Flexible mindset Acceptance of change Stress inoculation Meaning making Knowing who you are/strengths use
6. Fast SPARK	Reversed SPARK for stressful situations Knowledge: Taking notice Reaction: Refraining from action Affect: Emotion regulation Perception: Thinking about an action plan
7. Resilience muscles	Positive perception Positive emotions Positive relationships Knowing that you can/self-efficacy
8. Team resilience	SPARK Resilience for Teams model

The final three sessions are devoted to helping participants apply these skills in their workplace context. Session 6 helps the participants to select personally relevant techniques that can be used under pressure. It introduces KRAP, an inversed SPARK tool using the “Knowledge–Reaction–Affect–Perception” sequence based on the premise that in stressful situations emotional regulation should precede cognitive reframing [47]. Session 7 aims to develop prevention strategies (Resilience muscles), such as fostering positive emotions, investing in positive relationships by practicing forgiveness, altruism, and gratitude, and developing self-efficacy. The final session introduces team resilience factors that contribute to work resilience over and above the individual ones.

Every session begins with a brief introduction and a voluntary review of homework from the previous session followed by a 5-minute inclusion exercise (e.g., a quiz, a question to share responses or a brief guided positive psychology intervention). Next, the topic of the daily session and associated strategies are introduced. Each session normally includes 1 to 3 small-group sessions with 3 to 6 participants per group taking 30 to 45 minutes altogether. At the end, a 15-minute mindfulness exercise appropriate to the session’s topic is performed.

### Aim of the present study

The present study aimed to investigate the effectiveness of the SPARK Resilience Programme in an online (distance-based) setting using a mixed-method approach to inform the future adaptation of the program to a distance-based format.

## Methods

### Participants and procedure

The study used a two-group non-randomised controlled design with a pre-test and a post-test. The data were collected in April 2020. The participants were French-speaking volunteers who enrolled in the SPARK Resilience Programme. The intervention was delivered in a group coaching format via synchronous video conferencing using Zoom software over eight 90-minute sessions spanning four weeks. The sessions included a combination of teaching, whole-group interactions, questioning, using voice and chat functionalities, quizzes, small virtual group discussions, peer coaching, guided mindfulness exercises, and non-mandatory homework. The SPARK sessions were conducted by two trainers with postgraduate degrees in psychology (MSc and PhD, respectively), background in positive and organizational psychology, and extensive experience in group facilitation and resilience work.

The participants were recruited from among the individuals who had registered for a free pilot trial of the SPARK Resilience Programme for French-speaking adults scheduled to take place in April 2020. The training was advertised using mailing lists and positive psychology-themed websites in March. Although the advertisements mainly targeted working professionals, students were also allowed to register. Once it became clear that the number of potential participants would exceed the limitations imposed by the Zoom platform (*N* = 300), a second wave of the training was scheduled to take place in June 2020 in order to accommodate those who registered later. The data were collected in April, with the intervention and the wait-list control group comprised of April and June wave participants, respectively.

The week before the April wave was due to start, all SPARK participants from both groups received an email invitation to take part in a research study of psychological factors and dynamics of resilience in the context of the COVID-19 pandemic. The study involved completing a set of online questionnaires twice, at a one-month interval. Those who opted to take part in the study received a link to the pre-test questionnaire immediately and another link to the post-test questionnaire one month later. Participation in the study was voluntary and was not a condition for participation in the SPARK programme itself. The trainers had no information about the study participation or the responses provided by specific individuals. To ensure a realistic representation of SPARK programme participants, the study did not use any specific exclusion criteria. The research was conducted in accordance with the Declaration of Helsinki. Informed consent in an electronic form was obtained from all participants. The study protocol was approved by the HSE University Research Ethics Committee.

The number of questionnaires collected was 390 for the pre-test (177 in the intervention group and 213 in the control group) and 232 for the post-test (140 in the intervention group and 92 in the control group). For the present study, we used the data of participants who completed both the pre-test and the post-test and whose questionnaires could be matched based on the identifiers they provided. The resulting sample included 101 participants in the intervention group and 86 in the control group (three control group participants provided incomplete questionnaires; we report the effective sample size for each analysis).

The participants were mostly female (87.1%) and ranged in age from 20 to 76 (*M* = 46.9, *SD* = 10.8, median = 48, quartile range: 41–54). A majority of the participants had a Master’s degree or equivalent (64.0%) or a university degree (30.6%). Most participants were married or in a relationship (75.8%) and living together with other people during the confinement (87.6%). In terms of professional status, most were currently active: employed full-time (35.3%), self-employed (23.4%), business owners (15.8%), employed part-time (12.0%) or looking for a new job (6.5%).

### Instruments

***Positive and Negative Affect Schedule (PANAS)*** [48, 49], a 20-item list of adjectives reflecting positive (sample items: “enthusiastic”, “active”, “inspired”) and negative (“upset”, “guilty”, “irritable”) affective states. The participants were asked to evaluate the extent to which they experienced each of these feelings “over the past few days” using a 5-point Likert scale from 1 “Very slightly or not at all” to 5 “Extremely”.

#### Resilience scale

We used 7 items theoretically informed by the two-facet general definition of resilience as a combination of capacity for recovery (“I am able to adapt to change”, “I tend to bounce back from illness or difficulties”, “I can cope with any unexpected event”) and resistance (“Under pressure, I concentrate and think clearly”, “I am not easily discouraged by failures”, “I think of myself as a strong person”, “I am able to tolerate unpleasant feelings”). The participants were asked to evaluate the items using a 5-point Likert scale from 1 “Completely disagree” to 5 “Completely agree”. CFA has supported a theoretically expected single-factor structure (X^2^(14) = 25.21, *p* = .033; CFI = .982; RMSEA = .047, 90% CI [.014, .077]; SRMR = .053), the standardized factor loadings ranged from .40 to .65.

#### Personal meaning scale

This brief scale was validated as part of Positive Organisational Profile [50], a comprehensive French instrument measuring different aspects and predictors of well-being in organisations. It includes 4 items rated on a 7-point Likert scale (from 1 “Completely disagree” to 7 “Completely agree”): “I experience much joy in life and appreciate its every moment”, “Recently, my life seems to me rather monotonous and boring” (reverse-scored), “Generally, I feel that what I do in my life is useful and meaningful”, and “My daily activities often seem trivial and unimportant to me” (reverse-scored). CFA has supported a single dimension (X^2^(2) = 4.02, *p* = .134; CFI = .998; RMSEA = .054, 90% CI [.000, .131]; SRMR = .027) with item loadings in the .59-.84 range.

#### Perceived Stress Scale (PSS) [51, 52]

We used the 10-item version of this instrument (PSS-10) with a 5-point Likert response scale going from 1 “Never” to 5 “Very often”. We asked the participants to evaluate their experiences during the past week. Sample items: “…have you felt that you were unable to control the important things in your life?”, “…have you felt difficulties were piling up so high that you could not overcome them?”

#### Work Engagement Scale

This original 9-item French-language scale was validated as part of Positive Organisational Profile [50]. It was inspired by Schaufeli’s [53] model of work engagement with 3 dimensions, vigour (reflecting a feeling of energy at work: “My work gives me energy and inspires me”), dedication (reflecting interest in and commitment to one’s work: “My work is an important part of what I am, a vocation”), and absorption (reflecting an intense concentration on one’s work: “When I am working, I tend to forget about the things happening around me”). The items were evaluated on a 7-point Likert scale from 1 “Never” to 7 “All the time (every day)”. The theoretical bifactor model with a single dimension and three subdimensions was supported by CFA (X^2^(18) = 22.43, *p* = .213; CFI = .990; RMSEA = .030, 90% CI [.000, .064]; SRMR = .030). This scale was only administered to those individuals who reported being professionally active at the moment (*N* = 74 in the intervention group and *N* = 60 in the control group), i.e., were not students or temporarily unemployed.

The above-listed measures were used at both pre-test and post-test in both groups. Additionally, we asked several questions to the intervention group participants at post-test:

***Adherence to the program*** was monitored using two questions: “At how many of the 8 sessions of the SPARK Resilience Programme were you present?” and “How many times did you complete the ‘home assignments’ / intersession exercises? Please respond honestly”.

***Program satisfaction*** was measured using a brief satisfaction survey with 10 items asking to evaluate satisfaction with the program content, methods, tempo, teaching methods, teaching process, as well as the Zoom platform, the facilitators, the exchanges in the group, the help in case of difficulties, and, finally, overall satisfaction. The Likert response scale used 4 options, from 1 “Not satisfied at all” to 4 “Very satisfied”.

***Qualitative descriptions*** were obtained from the participants taking the intervention (both in April and June waves) using three open-ended questions: “How did you experience the SPARK Resilience program?”, “How could we improve the program? What did you appreciate most?”, and “Would you like to share something else with us?”.

### Data analysis

#### Quantitative analyses

To investigate the differences between the groups in the change from pre-test to post-test, we used univariate ANCOVA to compare the scores in the two groups at post-test, controlling for the pre-existing individual differences (i.e., pre-test scores) [54]. This was followed by simple effects analyses using Student’s t-test to explore the score change in each group. Next, we used multiple regression to find out whether demographic variables and adherence to the program could explain the individual differences in the effectiveness of the program within the intervention group. Finally, we explored the dimensionality of the program satisfaction items using principal component analysis and investigated the distribution of the resulting satisfaction scores. The analyses were performed in Jamovi 1.8.0. In line with the recent recommendations, the interpretations of the findings rely on effect sizes and their respective confidence intervals [55], but we also report exact significance levels for reference.

#### Qualitative analyses

The answers to the three open-ended questions submitted by participants (from both the April and the June waves) were combined and analysed using thematic analysis [56]. Given that all the collected feedback was in French, the analyses were carried out by two bilingual researchers who translated the participants’ quotes into English. First, the accounts were carefully read through to identify the main recurrent themes. Next, the accounts were analysed top-down into the six categories identified. Coded statements could consist of parts of sentences, full sentences or multiple sentences. Each statement could be coded once or twice, with an overlap in codes possible. The coding was performed by one expert and moderated by another expert; the discrepancies were resolved jointly.

## Results

### Quantitative findings

The descriptive statistics for both groups and the results of hypothesis tests are given in Table 2. Student’s t-test did not reveal any significant differences between the mean scores of the two groups at the pre-test. The two groups did not differ in age either. However, in the intervention group there was a higher proportion of female participants (93% against 80% in the control group, χ^2^(1) = 7.02, *p* = .008, Cramer’s *V* = .194), as well as of participants with a Master’s degree or above (73% against 53% in the control group, χ^2^(1) = 8.27, *p* = .004, Cramer’s *V* = .211).

**Table 2:** Descriptive statistics.

	α	Group	N	Pre-test	Post-test	d [95% CI]	d_ppc2_
				M (SD)	M (SD)		
Resilience	.76	Intervention	101	3.76 (0.55)	3.97 (0.52)	.48* [.27; .68]	.53
		Control	84	3.83 (0.52)	3.76 (0.55)	-.19 [-.41; .03]	
Positive Affect	.83	Intervention	101	3.40 (0.53)	3.68 (0.53)	.51* [.29; .71]	.56
		Control	85	3.35 (0.67)	3.29 (0.66)	-.10 [-.31; .12]	
Negative Affect	.84	Intervention	101	1.98 (0.53)	1.73 (0.47)	-.47* [-.68; -.26]	-.35
		Control	85	1.92 (0.57)	1.87 (0.63)	-.11 [-.32; .11]	
Meaning	.78	Intervention	101	5.56 (1.12)	5.90 (0.95)	.44* [.23; .65]	.40
		Control	84	5.28 (1.09)	5.17 (1.08)	-.12 [-.34; .09]	
Perceived Stress	.84	Intervention	101	2.47 (0.60)	2.18 (0.56)	-.45* [-.65; -.24]	-.52
		Control	85	2.32 (0.65)	2.36 (0.71)	.06 [-.15; .27]	
Work Engagement	.90	Intervention	74	5.91 (0.86)	5.96 (0.85)	.09 [-.14; .31]	.21
		Control	60	5.73 (0.94)	5.59 (1.08)	-.26 [-.51; .00]	

Note.

* Student’s paired-samples t-test *p* < .001. *d*–Cohen’s d for the difference from pre-test to post-test; *d*_ppc2_ –unbiased estimate of effect size for pretest-posttest control group designs [57].

According to ANCOVA, the group differences in change from pre-test to post-test were statistically significant for all 6 dependent variables. The strongest effects were observed for meaning (*F*_1,181_ = 25.79, *p* < .001, η^2^_p_ = .125, ω^2^ = .064), positive affect over the past few days (*F*_1,183_ = 24.45, *p* < .001, η^2^_p_ = .118, ω^2^ = .081), and resilience scores (*F*_1,182_ = 21.11, *p* < .001, η^2^_p_ = .104, ω^2^ = .055), indicating a more positive change from pre-test to post-test in the intervention group, compared to the control group. The effects for perceived stress over the past week (*F*_1,183_ = 9.78, *p* = .002, η^2^_p_ = .051, ω^2^ = .034), negative affect over the past few days (*F*_1,183_ = 6.15, *p* = .014, η^2^_p_ = .033, ω^2^ = .019), and work engagement (*F*_1,131_ = 4.84, *p* = .030, η^2^_p_ = .036, ω^2^ = .010) were more modest in magnitude, but also statistically significant. All the effects went in the direction consistent with the hypothesized positive effects of the intervention.

The simple effects analyses using paired-samples Student’s t-test revealed a statistically significant (*p* < .001) increase in positive affect, resilience, and meaning, as well as a decrease in perceived stress and negative affect in the intervention group. At the same time, there were no significant changes in the wait-list control group. For work engagement, we observed no significant change in the intervention group, but there was a marginally significant (*d* = -.26, *p* = .051) decrease in the control group over the study period.

Analyses of potential moderators of the intervention effects using the data from the intervention group only have revealed some individual differences. Controlling for perceived stress at baseline, age has emerged as a negative predictor of perceived stress at post-test (*b* = -.014, β = -.232, 95% *CI* [-.411, -.053], *p* = .012), suggesting that the effect of the intervention increased with the age of participants. A similar effect of age was observed for negative affect (*b* = -.011, β = -.226, 95% *CI* [-.401, -.052], *p* = .012), but not for any other dependent variables. Given these findings, we repeated the ANCOVA analyses with participant age as an additional covariate. The results were substantially the same for all dependent variables with only a marginal increase in effect sizes (not exceeding .006 for partial eta-squared and .004 for omega-squared). Education, family status, and the fact of living alone during the confinement did not explain any individual differences in the intervention outcomes.

The adherence to the intervention was fairly good: the intervention group participants reported attending, on average, 7.55 (*SD* = .80) out of 8 sessions with a minimum of 4 sessions. Only 8 participants (8%) have skipped 2 or more sessions. The number of sessions attended did not predict any individual differences in the intervention outcomes. The number of completed home assignments reported by intervention group participants at post-test has shown a larger individual variance, ranging from 0 to 7 (*M* = 3.76, *SD* = 1.98). However, the number of home assignments completed did not predict any differences in change at the post-test either.

The satisfaction survey was completed by 98 out of 101 intervention group participants. Parallel analysis [58] and the scree plot revealed a single dimension with component loadings ranging from .48 to .81 for individual items. The overall satisfaction rating calculated as a mean of the 10 satisfaction items (α = .85) was quite high (*M* = 3.81, *SD* = 0.27, range 2.7 to 4.0). The cumulative proportion of responses reflecting satisfaction (3 “Satisfied” and 4 “Very satisfied”) ranged from 96.9% to 100% across the items, indicating a high degree of participant satisfaction.

### Qualitative data

The qualitative accounts were provided by 151 individuals from both groups who took part in the intervention either at Wave 1 or at Wave 2 (following the wait-list period). Based on coding, we identified six main themes: solution focus/action orientation, awareness of emotion-cognition interaction, growth after adversity, emotion regulation strategies, mental health, and relationships and communication (Table 3).

**Table 3:** Thematic analysis summary.

Code	N	%
Solution focus/action orientation	111	73.50
Awareness of emotion-cognition interaction	102	67.50
Growth from adversity	95	62.91
Emotion regulation strategies	86	56.95
Mental health	76	50.33
Relationships and communication	74	49.00

***Solution focus/action orientation*** is a theme uniting action urges reflecting participants’ focus on setting goals and identifying solutions to current problems. This appears to be the most significant impact area of the SPARK resilience training, as this theme was most frequently mentioned. The majority of participants expressed a desire to implement what they had learned and experienced during the training program, either to influence their own life or someone else’s life. For example, one participant shared that they were now “Finding motivation and energy to carry out daily activities and those related to work”, whilst another said: “I have found my motivation, my energy, my dynamism, my ‘feeling capable of’”. Another participant said, “I am focusing on what I really want to do and how I want to do it, how I can become the best possible me whilst staying aligned with my values”. Participants also reported having identified practical, personalised tools to implement in their personal and professional life and being able to set goals for the future. A love of learning, accompanied by a renewed motivation, “a new intellectual motivation”, and a desire to try out new possibilities were common: “I decided to take a distance learning option and finish the training in hypnosis that I had started, and to follow the SPARK resilience training to become a trainer later on”.

#### Awareness of emotion-cognition interactions

Developing an understanding of the SPARK model involves identifying the situation, perception, affect, reaction, and knowledge in application to real-life situations. This theme reflects participants’ experiences of being aware of the interaction between the way one interprets events and the subsequent associated emotions, as well as of the mechanisms underlying this connection and of one’s own cognitive biases. It also refers to practicing cognitive flexibility, creating a distance from a difficult situation by reflecting on it and contextualising it. For instance, one of the participants described this distancing effect as follows: “I have learnt to accept uncertainty, to look at the glass half full compared to the glass half empty, and am now able to see how my own thoughts can impact the way I feel”, whilst another said: “What I thought was ‘failure’ may not be that much of a bad thing”. Other participants described how they could now link the different SPARK components, for instance, a stressful situation (“I had a burnout one year ago in my professional life”) with its perception (“I thought it was not possible to get away from this work overload of and being in conflict with my value”) and affect (“this state has affected me on all levels: emotional, psychological, physical and existential”). Similarly, some participants described using their understanding of resilience to work on their perceptions and modify their ways of looking at things. As one participant expressed it, “I now analyse my challenges through the filter of SPARK”.

#### Growth from adversity

This theme unites participants’ experiences of their capacity to grow following challenging and painful incidents by finding meaning and by making sense of what happened. This process involves redefining one’s identity and self-perception after life-shaking events, as well as discovering and relying on one’s strengths to rise from these situations. For instance, a participant whose colleague had suddenly decided to stop their professional work alliance embraced the situation this way: “Finally, this was a very beautiful opportunity to feel fully at my place professionally”. Another participant related the way she had discovered her strengths and found her life meaning: “My career as a humourist was born! I had found a true meaning to my life. I wrote a play that I then performed on stage!”. In addition to discovering strengths, the process of growth from adversity can bring other benefits: the very way one views their relationship with themselves, with others, and with the world can be changed as part of this personal transformation process. As one participant described it, “I am realising the importance of the changes that happened to my values, to my way of looking at the world and at others”. Another person shared their new-found awareness of their own values, those being “authenticity, self-realisation, personal and professional accomplishments and recognition”.

#### Emotion regulation strategies

This theme comprises the experiences of using emotion regulation strategies to deal with difficult emotions recounted by participants. Strategies to regulate emotions involved a variety of helpful actions, such as expressing gratitude, listening to music, meditating, making a pause in one’s work day, and looking at the glass as half full rather than half empty. Participants shared: “Practicing a mindfulness exercise that involved singing negative thoughts was a great discovery that I will use again in the future” and “I am now taking the time to notice the positive side of a situation every day”. Comments expressing the importance of positivity in dealing with difficult emotions were common. Emotional regulation strategies used by participants also involved disputation, de-catastrophising, reappraising, reframing, and using self-compassion: “if you fail, tell yourself that you were not in the best conditions to accomplish this task”. Naming and identifying emotions was also one of the strategies participants used to manage and soothe difficult emotions: “Reconnecting with emotions, even with the most unpleasant ones, is essential in allowing an optimal development, in adapting to different situations encountered, in acknowledging and responding to our psychological needs, in enjoying fully the experiences that we are living, in order to spend less time in the autopilot mode, and be more aware of our internal states. This leads to being able to respond in a relevant way to the demands of a situation instead of being in reaction mode”. In addition, practical and simple actions such as cooking, writing, savouring, learning new skills, physical activity, cultural activities, nature, praying, nutrition, health care, and satisfying basic needs, such as eating when hungry and sitting down when tired, were all mentioned as being helpful for participants.

#### Mental health

About half of the participants observed that their mental health was enhanced by SPARK resilience training. In particular, resilience and psychological resources appeared to have been strengthened, with participants expressing feeling stronger, more loving, and having more dignity and hope. For instance, one participant shared: “I was trying to get back up the slope, to take care of myself to regain some energy and dignity”. Another said: “I felt more able to resist circumstances and this has helped my anxiety”. Increased awareness of one’s perceptions, emotions and needs might lead to positive mental health changes. Several participants reported an improvement in their levels of emotional well-being. As one participant recounted, “Even with everything that is currently going on in the world, I feel more content and stable in myself”; another person expressed feeling “liberated” after a job interview that did not lead to the job; yet another participant stated: “I am proud for having acted upon my needs and not based on my father’s thoughts of what he considers as normal. Each of us has our own way of doing things, so I want to continue listening to myself.” In addition, participating in the SPARK training seems to have enhanced feelings of psychological safety and grounding for some, referred to by one participant as “a sense of internal security”.

#### Relationships and communication

Finally, the development of positive relationships and more effective communication with others emerged as one of the results. Some participants have experienced being more able to receive and welcome help from others, for example: “I have found people on my way on whom I could rely and I am now surrounded by a stable affective environment” or “I have received so much help and support from my father-in-law”. Reaching out to others was also common among participants, with some reporting having sought support from others in their group: “I felt the need to continue to exchange with [another participant’s name] who gave me advice when working in a small group”. Another participant shared: “Even though we did not know each other well, the intimacy developed in breakout rooms was simply incredible”. Participants reported developing more positive expectations towards others and being on the giving end of kindness and altruism: “I devote myself now to trying to help others by transmitting my experience, my learning and discoveries”, “I make myself available and listening to my close people, my clients, my friends”. Some participants have experienced relationships to be helpful and strengthened when confronting adverse situations. This was not limited to friends and family: supportive positive work relationships were also described. For instance, a participant expressed the following regarding her relationships with her colleagues after dealing together with a difficult manager: “Our relationships became more fluid, with more sharing, and a lot more trust between us”.

## Discussion

Although the SPARK Resilience programme has been evaluated in quantitative studies using adolescent samples [43–45], no previous research has looked at its effects in adult samples. The present study aimed to extend the previous findings by testing the intervention in a sample of French adults and by using a mixed-methods approach to explore the effects of the programme based on qualitative feedback from participants. An additional strength of the study is its realistic setting of a looming threat of the developing COVID-19 pandemic, when the participants were facing the unprecedented experience of a lockdown, trying to adapt their work and life routines.

The quantitative findings that we observed are in line with those obtained in previous studies. Pluess and colleagues [44] using a 12-week SPARK intervention in an adolescent British sample found small beneficial effects on resilience (*d* = .31) and depression (*d* = -.21) outcomes. Kibe and colleagues [45] found a significant effect of the intervention on generalized self-efficacy in Japanese adolescents but did not discover any statistically significant effects on depression, self-esteem or resilience, although the results suggested significant individual variance in the intervention outcomes for the latter two variables.

The effects that we observed in the present study are not only statistically significant but also stronger in magnitude compared to the previous studies of the SPARK Resilience Programme with *d* in the .44-.51 range in the intervention group for all variables, except for work engagement. This is hardly surprising, given that resilience interventions tend to have stronger effects in adults, compared to child and adolescent samples [41]. The values of partial eta-squared correspond to *r* in the .18-.35 range, indicating small to medium effect sizes typical for resilience interventions in low-risk populations [41]. According to the d_ppc2_ effect size measure, the effects on resilience, positive affect, and perceived stress are above the threshold (*g* = .41) proposed to define the effects of potential practical significance [59]. Given the persistent challenge of the ongoing pandemic, the weaker effects of the intervention on negative affect and work engagement that were being affected by the ongoing lockdown are hardly surprising.

The findings concerning individual differences in the intervention outcomes suggest that SPARK resilience training was more effective at reducing stress and negative affect for older adults. (An investigation of the scatterplots suggested a linear association of the intervention effectiveness with age; unfortunately, the sample was too small to allow sufficient power for a statistical analysis of the differences across age groups). On the one hand, this finding could be explained by the fact that the health risks associated with the COVID-19 pandemic are vastly different across age groups, given that existing meta-analyses indicate stronger effects of resilience interventions in populations at higher risk [41]. On the other hand, older adults might differ in the quality of motivation or in the development of personality resources, such as self-regulation or reflective processes, that could predict individual differences in the outcomes of positive interventions [60]. Future research needs to replicate this finding outside the context of the COVID-19 pandemic, controlling for baseline personality resources.

The data showing high participant adherence and satisfaction indicate that the intervention was extremely well received, corroborating the principal study outcomes. This result could also be explained by the pandemic context: after all, the study was conducted during the very early period of lockdown, which was first introduced in France on March 17. Based on data from a representative French sample, March and April 2020 were characterized by higher levels of negative affective experiences, such as anxiety, loneliness, boredom, and depression, compared to subsequent months (see the data visualisation tool presented by Leander and colleagues [61]). During this time, many individuals were facing the new risks and the uncertainty associated with COVID-19, as well as the new challenges associated with the transition to distance-based work and study, that could all increase stress levels [62, 63]. This was exactly the time when people were most likely to be in immediate need of psychological support and when a positive psychological intervention helping them to activate and build their coping resources, as well as to offer and receive social support, was most welcome.

The qualitative data extend the quantitative results by shedding light on the specific benefits and areas of improvement resulting from the SPARK intervention in the pandemic context. Some of the emerging themes, such as awareness of cognition-emotion interactions, development of the repertoire of emotion regulation strategies, as well as strengthened positive relationships, are generally consistent with the previous qualitative findings in an adolescent sample [44]. Other themes, such as growth from adversity, focus on mental health, and action orientation reflecting the need to find motivation and energy to go on, might reflect the specific positive effects of the intervention in the context of an ongoing pandemic. In short, these qualitative results can be seen as a case study showing how the SPARK Resilience Programme can help people to discover the resources needed to face an unprecedented and very challenging situation.

### Limitations

Obviously, the study is not without its limitations. The first limitation is its modest sample size. According to sensitivity analyses, the resulting sample has allowed to attain sufficient power (.80) for small to medium-sized effects of the intervention (η^2^ = .040). However, the sample size for moderator analysis involving only the intervention was relatively small, only allowing to gain acceptable power for stronger effects (*f*^2^ = .08), which does not allow to rule out the possibility of other variables moderating the intervention effectiveness.

Second, the sample was not representative and heterogeneous with respect to employment status and age. Also, the prevalence of female participants did not allow us to explore the gender differences in the intervention outcomes that have been reported earlier [43, 45]. Future studies using larger and more diverse samples could reveal more individual differences in the response to SPARK Resilience training.

Third, the lack of proper randomization resulted in non-equivalent groups. Nevertheless, the use of a pre-test/post-test research design has allowed us to control for baseline differences in psychological variables. Randomization would have meant withholding the possibility to obtain psychological support from some people who may have needed it most during the very beginning of the lockdown. This is why we opted to form the intervention group on a first-come first-served basis until the maximum size of the group the facilitators could accommodate was reached. In the end, all the study participants had the opportunity to benefit from the intervention.

Fourth, at the time when the study was conceived, we were not aware of the existence of any validated French versions of brief instruments to measure resilience, such as the BRS-F [64] or RS-14 [65]. Future studies could improve the outcome measure validity by using these newer instruments.

Finally, the absence of a placebo or an alternative intervention does not allow to rule out participant expectation effects and the absence of a passive control group did not allow for a longer-term follow-up to see if the intervention effects were sustainable. However, a different study design would be associated with additional ethical and organizational challenges too difficult to resolve quickly during an ongoing pandemic. Nevertheless, we believe that the findings constitute strong evidence in favour of important immediate effects of SPARK Resilience training in a challenging context.

## Conclusion

Psychological science tells us that resilience can be developed, with evidence pinpointing to multiple resources that can be built through deliberate coaching, training, and positive interventions. SPARK Resilience can be used as a brief coaching model, but also as a structured coaching approach to organise work with individuals, groups of employees or even teams. This approach is flexible enough to grow and develop in line with new research and practice discoveries.

SPARK Resilience training already benefits from a substantial evidence base constituted not only by the scientific studies of the mechanisms and processes of resilience, but also by empirical validation under various conditions. The present study shows the efficacy of the program in a French adult sample during the very early stage of the COVID-19 pandemic, contributing to the body of knowledge showing how positive psychology can help people to remain efficacious and to stay well even under such unprecedented challenges.

## Supporting information

S1 Data (XLSX)
